# Comparative Analysis of Physiological Responses and Intestinal Microbiota in Juvenile Soft-Shelled Turtle (*Pelodiscus sinensis*) Fed Four Types of Dietary Carbohydrates

**DOI:** 10.3390/ani14121781

**Published:** 2024-06-13

**Authors:** Haoran Sun, Yue Zhang, Tiancong Ren, Qian Gao, Xueying Shi, Xiangce Li, Peiyu Zhang, Zhi Li, Haiyan Liu

**Affiliations:** 1 Hebei Key Laboratory of Animal Physiology, Biochemistry and Molecular Biology, College of Life Sciences, Hebei Normal University, Shijiazhuang 050024, China; sunhr99@163.com (H.S.); 17331027858@sina.cn (Y.Z.); rtc1991@163.com (T.R.); gao7ian@163.com (Q.G.); shixueying@stu.hebtu.edu.cn (X.S.); xiangceli@163.com (X.L.); peiyuz@hebtu.edu.cn (P.Z.); 2 College of Resource and Environment Sciences, Shijiazhuang University, Shijiazhuang 050035, China; 3 Hebei Collaborative Innovation Center for Eco-Environment, Shijiazhuang 050024, China

**Keywords:** *Pelodiscus sinensis*, carbohydrate, growth, glucose and lipid metabolism, intestinal microbiota

## Abstract

**Simple Summary:**

Carbohydrate is an important energy nutrient in the feed of aquatic animals. Generally, aquatic animals usually exhibit a varying efficiency in utilizing different carbohydrate sources. In order to understand the carbohydrate utilization efficiency, this study investigated the physiological responses of soft-shelled turtles (*Pelodiscus sinensis*) that were fed four types of carbohydrates with different complexities and configurations. The results indicated that the best growth performance and feed efficiency were found in the starch diet, followed by the glucose, fructose, and cellulose groups in sequence. Dietary starch demonstrated a robust lipogenic function by inducing the expression of the genes involved in glucolipid metabolism, with the results of elevated plasma triglyceride levels and an increased lipid content in both the whole body and the liver. Glucose and fructose diets caused postprandial hyperglycemia in *P. sinensis* due to the un-inhibited gluconeogenesis. *P. sinensis* that were fed a fructose diet did not exhibit a higher lipid deposition compared to the glucose diet, as seen from mammals. Cellulose was not a suitable energy source for *P. sinensis*.

**Abstract:**

A 60 day feeding trial was conducted to evaluate the impacts of dietary carbohydrates with different complexities and configurations on the growth, plasma parameters, apparent digestibility, intestinal microbiota, glucose, and lipid metabolism of soft-shelled turtles (*Pelodiscus sinensis*). Four experimental diets were formulated by adding 170 g/kg glucose, fructose, α-starch, or cellulose, respectively. A total of 280 turtles (initial body weight 5.11 ± 0.21 g) were distributed into 28 tanks and were fed twice daily. The results showed that the best growth performance and apparent digestibility was observed in the α-starch group, followed by the glucose, fructose, and cellulose groups (*p* < 0.05). Monosaccharides (glucose and fructose) significantly enhanced the postprandial plasma glucose levels and hepatosomatic index compared to polysaccharides, due to the un-inhibited gluconeogenesis (*p* < 0.05). Starch significantly up-regulated the expression of the genes involved in glycolysis, pentose phosphate pathway, lipid anabolism and catabolism, and the transcriptional regulation factors of glycolipid metabolism (*srebp* and *chrebp*) (*p* < 0.05), resulting in higher plasma triglyceride levels and lipid contents in the liver and the whole body. The fructose group exhibited a lower lipid deposition compared with the glucose group, mainly by inhibiting the expression of *srebp* and *chrebp*. Cellulose enhanced the proportion of opportunistic pathogenic bacteria. In conclusion, *P. sinensis* utilized α-starch better than glucose, fructose, and cellulose.

## 1. Introduction

The soft-shelled turtle (*Pelodiscus sinensis*) is an aquatic reptile found in China, Japan, and South Korea. Its natural population has been steadily decreasing, leading to its assessment as a vulnerable species on the IUCN (International Union for Conservation of Nature) Red List [1]. In recent years, *P. sinensis* has emerged as a prominent freshwater aquaculture species in China due to its nutritional value, pharmacological functions, and immune-boosting properties [2]. In 2022, China achieved a substantial yield of 373,709 metric tons of *P. sinensis*, as indicated by the latest statistical data [3]. As a carnivorous animal, *P. sinensis* primarily consume a varied diet in their natural habit, including small fish, mollusks, insect larvae, and the seed of marsh plants [4]. The rapid growth of *P. sinensis* obtained in intensive aquaculture heavily relies on the dietary protein supply, primarily sourced from fishmeal [5,6]. But the continually lower availability and the increasing price of fishmeal have become the bottleneck for the sustainable development of turtle aquaculture. Hence, some strategies should be implemented to reduce the protein levels and substitute fishmeal with alternative nutrients in the artificial feed of turtles. Carbohydrates are the second most important energy source in turtle feeds, offering numerous benefits such as excellent accessibility, cost-effectiveness, favorable feed shaping, and the absence of ammonia emissions [7,8,9,10]. Understanding the utilization efficiency of carbohydrate in the feed of *P. sinensis* is helpful to resolve this problem.

Glucose is a common monosaccharide in nature and serves as the dominant carbohydrate source for metabolic processes in the animal body. Starch, polymerized from glucose, is the main complex carbohydrate source stored in plant. Compared to monosaccharides, polysaccharides are better utilized for many aquatic animals, including *P. sinensis* [11,12,13,14,15,16,17]. Nevertheless, some aquatic animals exhibited a better utilization capacity of monosaccharides [18,19,20]. The discrepancies in the utilization ability of various carbohydrate sources are usually considered to be closely related to the complexity of the carbohydrate structure, which directly influences both the speed of carbohydrate absorption in the intestine and postprandial hyperglycemia. The utilization capacity of carbohydrates with different complexities varies in different species, and may be correlated to the digestibility and metabolism. A recent study demonstrated that the Nile tilapia (*Oreochromis niloticus*), which fed on a polysaccharide diet, manifested better lipid deposition and a higher expression of key genes in glycolysis and lipid metabolism than monosaccharide manipulation [17]. Therefore, more studies on digestibility and metabolism should be conducted to disclose the mechanism of utilizing carbohydrates with different complexities.

Fructose is a natural isomer of glucose, exhibiting a different metabolic pathway in the bodies of animals compared to glucose. In contrast to glucose, fructose metabolism is independent of insulin and is not regulated by the feedback of ATP and citrate (i.e., there is no rate-limiting enzyme in fructose metabolism), resulting in a very rapid metabolic process [21]. Research has demonstrated that fructose is more prone to promoting lipid deposition compared to glucose in mammals, and excessive fructose consumption would lead to hyperlipidemia and non-alcoholic fatty liver disease [22]. However, this phenomenon has not been observed in fish studies. Most fish that are fed on a fructose diet usually show a decrease or stabilization in triglycerides and body lipid content [9,23]. At present, the underlying mechanism for the differences in fructose metabolism between mammals and aquatic animals remains unknown. Additionally, the difference in glucose and fructose utilization has not been reported in *P. sinensis*. So, further investigations on fructose supplementation in the diet are necessary to address this knowledge gap. 

Intestinal microbiota occupies an essential position in maintaining the host’s nutrient metabolism and body health, and also participates in the digestion and fermentation process of dietary carbohydrates [24]. Different diets affect the structure of the intestinal microbial community in aquatic animals and an unbalanced diet composition may lead to the augmentation of harmful bacteria in the intestine, causing inflammation and intestinal damage [25,26]. Currently, the impact of carbohydrate sources on the intestinal microbiota of aquatic animals has received scant attention, despite the fact that this host–microbe interaction holds the potential to shed light on numerous unresolved questions.

Given the challenges in the feed of *P. sinensis* and advances in research on carbohydrate metabolism, we hypothesize that *P. sinensis* possess a distinct metabolic mechanism for utilizing various carbohydrate sources. Therefore, this study was conducted to investigate the impacts of four types of carbohydrates with varying complexities and isomers (glucose, fructose, α-starch, and cellulose) on the growth performance, physiological indices, digestibility, intestinal microbiota, and gene expression related to glucose and lipid metabolism in *P. sinensis*. The comprehensive research on the physiological responses to different types of carbohydrates would lay the foundation for enhancing dietary carbohydrate utilization and promoting the sustainable development of *P. sinensis* aquaculture.

## 2. Materials and Methods

### 2.1. Experimental Design and Breeding Process

Four isonitrogenous (48%) and isolipidic (8%) experimental diets with different carbohydrates were formulated by adding 170 g/kg glucose, fructose (monosaccharide), α-cassava-starch (digestible polysaccharide), or cellulose (indigestible polysaccharide), respectively (Table 1). The total dry feed ingredients were weighed, mixed, and crushed through 80 mesh screens, followed by supplementation with oil and water. Particles of 3.0 mm diameter were made using a feed pelleting machine (EL-260, Youyi Machinery Factory, Weihai, China) and were preserved in a refrigerator at −20 °C until use.

The turtles (approximately 3 g) used in this experiment were obtained from Yutian Farm (Tangshan, China). Acclimatization was conducted for two weeks under conditions suitable for the growth and survival of *P. sinensis*. After that, 280 turtles (5.11 ± 0.21 g) were randomly selected, averagely allocated to 28 tanks, and fed with the respective experimental diets in septuplicate to apparent satiation at 08:30 and 17:30 each day. After 30 min of feeding, the feed residue was siphoned out, dried, and weighed. The measured dissolution loss rate of feed was used to accurately calibrate the feed intake. During the experimental period, the culture water was monitored to maintain a suitable quality (temperature 30 ± 0.5 °C, pH 7.8 ± 0.2, dissolved oxygen concentration ≥6 mg/L, and ammonia nitrogen concentration ≤0.1mg/L). The feeding trial lasted for 60 days.

At the end of the feeding trial, turtles were deprived of diets for 24 h. All turtles were anesthetized with eugenol (1:10,000; Shanghai Reagent Corp., Shanghai, China) and were subsequently weighed in batch. Three turtles per tank were sampled randomly to analyze whole-body proximate compositions. Additionally, another three turtles from each tank were randomly chosen and individually weighed, and were then sampled for blood and other tissues. Blood was drawn from the neck and put in the centrifuge tubes with heparin rinse, and were centrifuged at 4 °C, 3000× *g* for 15 min to obtain plasma, which was preserved at −80 °C for further biochemical analysis. After blood collection, the turtles were dissected on an ice-cold plate for sampling viscera and liver to calculate the hepatic somatic index (HSI), visceral somatic index (VSI), proximate compositions of liver, and the intestinal substances were isolated under sterile conditions for intestinal microbiota analysis. The mid-gut and liver tissues (1 cm length) were taken and fixed in 4% paraformaldehyde solution waiting for morphological analysis. Subsequently, the remaining liver was wrapped in aluminum foil and quickly frozen in liquid nitrogen, then stored in a −80 °C refrigerator for further gene expression studies.

### 2.2. Apparent Digestibility Analysis

In order to determine the apparent digestibility, yttrium oxide (Y_2_O_3_) was supplemented at a 1 g/kg level in diets as an inert indicator. From the second week of formal trial, fresh feces (in a capsule state) in each tank was collected daily, dried at 65 °C for 12 h, and stored at −20 °C. The yttrium contents in feed and feces were measured using an inductively coupled plasma spectrometer (LABTAM 8410, Labtam Instrument Co., Ltd., Canberra, Australia). Meanwhile, the crude protein and lipid contents and energy values of feed and feces were determined. The apparent digestibility coefficients were calculated using the following method:

ADC (Apparent digestibility coefficient) of dry matter (%) = 100 × [1 − (Y_2_O_3_ in diet/Y_2_O_3_ in feces)];

ADC of nutrient or energy (%) = 100 × [1 − (Y_2_O_3_ in diet/Y_2_O_3_ in feces) × (nutrient or energy in feces/nutrient or energy in diet)].

### 2.3. Histological Analysis

The fixed intestinal tissues were dehydrated step-by-step with different concentrations of alcohol and xylene in a mixed xylene solution, which was followed by waxing, embedding, and slicing, as described by Guo et al. (2023) [27]. The intestinal sections were stained using hematoxylin and eosin (H&E). The fixed liver segments were stained using oil red O. All slices of intestinal sections and liver lipid droplets were observed with a ZEISS microscope (Imager A1m, Carl Zeiss AG, Oberkochen, Germany).

### 2.4. Proximate Composition and Plasma Biochemical Analysis

The nutrient compositions in the diets, turtle bodies, feces, and livers were determined using standard methods [28]. Moisture, crude protein, and ash contents were analyzed using an oven (GZX-9240MBE, Shanghai, China), Auto Kjeldahl System (KjeltecTM 8420, FOSS Tecator, Hillerød, Denmark), and muffle furnace (Taisete Co. Ltd., Tianjin, China), respectively. The crude lipid contents were determined using the Soxhlet extraction method. The moisture of the liver was analyzed using a vacuum freeze dryer (GZX-9240MBE, Shanghai, China), and the liver lipid content was determined following the chloroform–methanol extraction method [29], according to the description of Peng et al. (2014) [30]. Gross energy was measured using a Parr 6200 Calorimeter (Parr Instrument Company, Moline, IL, USA). Liver glycogen contents were determined according to the instruction of commercial kits (Nanjing Jiancheng Bioengineering Institute, Nanjing, China). Plasma glucose, total protein, cholesterol, and triglyceride contents were determined using the glucose oxidase method, biuret method, cholesterol oxidase method, and GPO-POD method, respectively.

### 2.5. Analysis of Intestinal Microbiota

Genomic DNA from the intestinal sample was extracted with the PowerFecal™ DNA Isolation Kit (MoBio Laboratories, Carlsbad, CA USA). The Illumina MiSeq platform was used to perform the high-throughput sequencing after amplification of the 16S rRNA V3-V4 region with barcoded fusion primers of 338F and 806R. All sequences were classified into operational taxonomic units (OTUs) picked at a 97% similarity level using QIIME 1.9.1 (Quantitative Insights Into Microbial Ecology) after the removal of low quality scores and were then merged using FLASH 1.2.11 software.

### 2.6. RT-qPCR Analysis

Total RNAs from intestinal and liver tissues were extracted with TRIzol reagent, according to the manufacturer’s instructions. The quantity and quality of the extracted RNA were analyzed at 260/280 nm using an ultrafine ultraviolet spectrophotometer (NanoDrop 1000, Thermo, Waltham, MA, USA). cDNA was synthesized via reverse synthesis using the PrimeScriptTM reverse transcription kit (TransGen Biotech Co., Ltd., Beijing, China). The cDNA concentrations were unified according to the concentrations determined before qPCR. A SYBR Green I SuperMix kit (TransGen Biotech Co., Ltd., Beijing, China) was used to determine the gene expressions. β-actin was adopted as the reference gene, and PCR was performed using real-time quantitative PCR. Specific Primers were designed with Premier 5.0 software (Table 2). RT-qPCR was performed on an ABI7300 qPCR system (ABI Prism 7300, Waltham, MA, USA) with a total volume of 20 μL, containing 10 μL of 2 × Trans-Script Top Green qPCR Super, 0.5 μL of 10 mM forward and reverse primers, 7 μL of nuclease-free water, and 2 μL of cDNA templates. The following reaction conditions were used: 94 °C for 30 s followed by 45 cycles consisting of 94 °C for 5 s and 60 °C for 30 s. After each reaction, a melting curve analysis of the amplification products was performed to determine the specificity of the amplification reaction. The relative expressions of the target genes were calculated according to the 2^−ΔΔCt^ method.

### 2.7. Statistical Analysis

All data are shown as mean ± S.E.M. Before analysis, the normality and homogeneity of variance were verified using a Shapiro–Wilk test and Levene’s test, respectively. One-way ANOVA was used in this study and was carried out using STATISTICA 10.0 software (Statsoft Inc., Tulsa, OK, USA). Whenever there was a significant difference, Duncan’s test was used for the multiple comparisons. Alpha-diversity and beta-diversity analysis of intestinal microbial communities were performed using QIIME. Beta diversity of microbial communities among samples was analyzed using UniFrac distance metrics and visualized via Principal Coordinate Analysis (PCoA). Statistical differences in the relative abundance of microbiota were analyzed using STAMP based on Welch’s *t*-test. A *p* < 0.05 was considered as the significant level.

## 3. Results

### 3.1. Growth Performance

Table 3 shows that the turtles fed with an α-starch diet had a significantly lower feeding rate (FR) and feed conversion ratio (FCR) than those fed other diets (*p* < 0.001). The values of FBW, WGR, and SGR in the starch diet were higher than that in the cellulose group (*p* = 0.024). The starch-fed turtles showed a significantly higher PER than the other groups (*p* < 0.001). There are significant differences in PDE or LDE values between any two groups, and the order is starch > glucose > fructose > cellulose (*p* < 0.001). The HSI and VSI of turtles fed glucose and fructose diets were dramatically higher than those in the starch and cellulose groups (*p* < 0.001).

### 3.2. Apparent Digestibility Coefficient

Different types of carbohydrates remarkably impacted the apparent digestibility coefficients of turtles (Table 4). The ADCs of dry matter, energy, and carbohydrates among all treatments exhibited significant differences between any two groups, following the order of starch > glucose > fructose > cellulose (*p* < 0.001). The turtles fed with glucose and α-starch had a significantly higher ADC_protein_ than those fed with fructose and cellulose diets (*p* = 0.001).

### 3.3. Proximate Compositions of Whole Body and Liver

Table 5 demonstrates that the crude protein and lipid contents of turtles were significantly different between any two groups, with the order of starch > glucose > fructose > cellulose (*p* < 0.001), but the moisture contents exhibited a complete reversal in comparison to the protein and lipid trends. The liver lipid levels in the glucose and starch groups were significantly higher than those in the fructose and cellulose groups (*p* = 0.001), and the liver glycogen contents of turtles fed with a cellulose diet was significantly lower than those fed with other diets (*p* < 0.001).

### 3.4. Plasma Biochemical Parameters

Table 6 shows that significantly higher plasma glucose levels were observed in glucose and fructose treatments compared to starch and cellulose diets (*p* = 0.002). There are significant differences in plasma triacylglycerol (TG) levels among the groups in the following order: starch > glucose > fructose > cellulose (*p* < 0.001). The plasma total cholesterol contents of turtles in the glucose and cellulose groups were remarkably lower than that in the fructose and starch groups (*p* = 0.006). Plasma total protein contents were not dramatically affected by carbohydrate sources (*p* = 0.386).

### 3.5. Intestine and Liver Tissue Morphology

**Figure 1:**
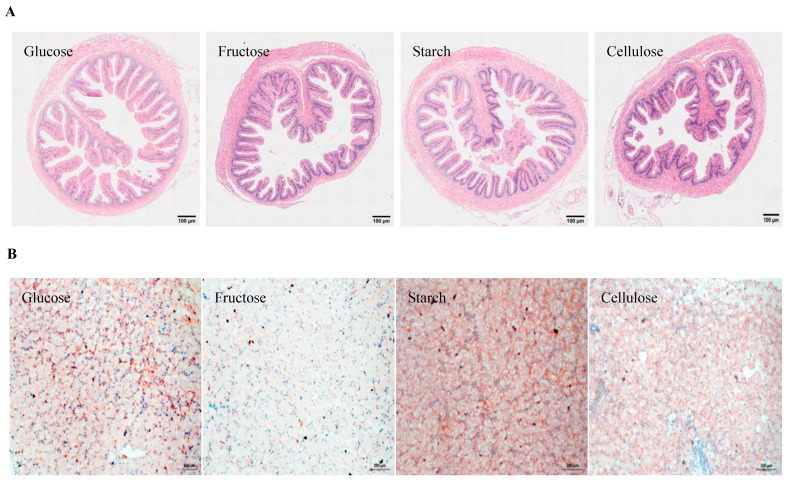
Histomorphological analysis of mid-gut and liver in *Pelodiscus sinensis* fed with diets containing different types of carbohydrate. (**A**) Mid-gut H & E stained sections. (**B**) Liver oil red O sections.

The intestinal tissue structures in all treatments were clear and complete. The intestinal villus of the turtles fed with a starch diet were slender and mostly finger-like, while the intestinal villus of the turtles fed with a cellulose diet were short and conical (Figure 1A). Oil red O staining of liver sections revealed a higher presence of lipid droplets in the starch and glucose groups compared to the fructose and cellulose groups (Figure 1B).

### 3.6. mRNA Expression of Genes Related to Glucose and Lipid Metabolism

**Figure 2:**
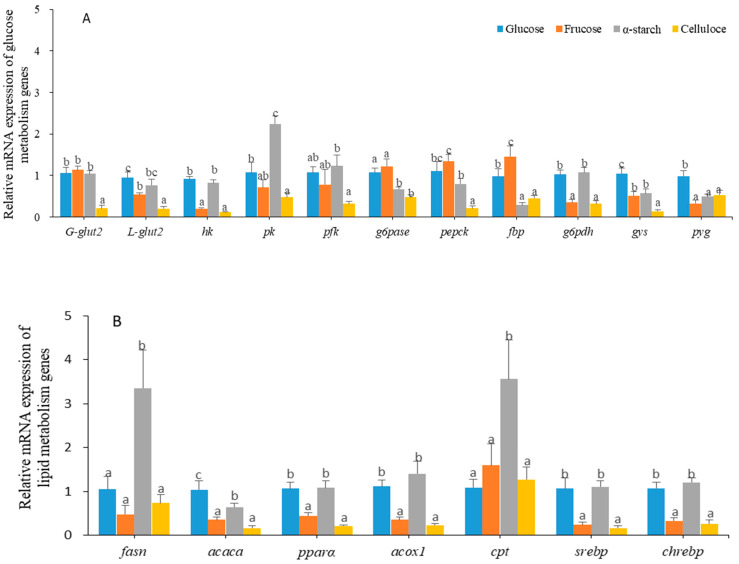
The mRNA expression of genes related to glucose and lipid metabolism. (**A**) Relative mRNA expression of glucose metabolism genes, glucose transport protein in gut and liver (G-*glut2* and L-*glut2*), glucose catabolism (*hk, pk*, and *pfk*), pentose phosphate pathway (*g6pdh*), gluconeogenesis (*pepck*, *fbp*, and *g6pase*), and glycogen synthesis and decomposition (*gys* and *pyg*). (**B**) Relative mRNA expression of lipid metabolism genes, lipid synthesis (*fasn* and *acaca*), lipid decomposition (*acox1*, *cpt*, and *pparα*) and transcriptional regulators (*srebp* and *chrebp*). Bars with different letters show a significant difference (*p* < 0.05) (*n* = 7).

The relative mRNA expression of genes related to glucose metabolism is depicted in Figure 2A. The expressions of *glut2* in the gut and liver of those in the cellulose group were significantly lower than that in other groups, while the liver *glut2* mRNA in the fructose group was down-regulated significantly compared to the glucose group (*p* < 0.001). Among all the groups, the gene expressions of the key enzymes involved in the glycolysis pathway were the lowest in the cellulose group. The *hk* expressions in the glucose group, as well as the *hk* and *pk* expressions in the starch treatment, were significantly elevated compared to the fructose group (*p* < 0.001). Genes related to gluconeogenesis in the fructose group exhibited the highest level of expression among these groups, and were significantly higher than that in the starch and cellulose groups. The *g6pdh* mRNA levels in the glucose and starch groups were dramatically higher than that in the fructose and cellulose treatments (*p* < 0.001). The expressions of *gys* and *pyg* in the glucose group were the highest levels among all groups.

The relative mRNA expressions of the genes involved in lipid metabolism are shown in Figure 2B. The expression of *fasn* in the starch group, as well as the expression of *acaca* in the glucose and starch groups, were significantly higher than that in the fructose and cellulose groups (*p* = 0.002, *p* < 0.001, respectively). The mRNA levels of *pparα*, *acox1*, *srebp*, and *chrebp* in the starch and glucose groups were significantly higher than that in the fructose and cellulose groups, with the *cpt* gene in the starch diet exhibiting the highest expression level among all treatments.

### 3.7. Intestinal Microbiota

**Figure 3:**
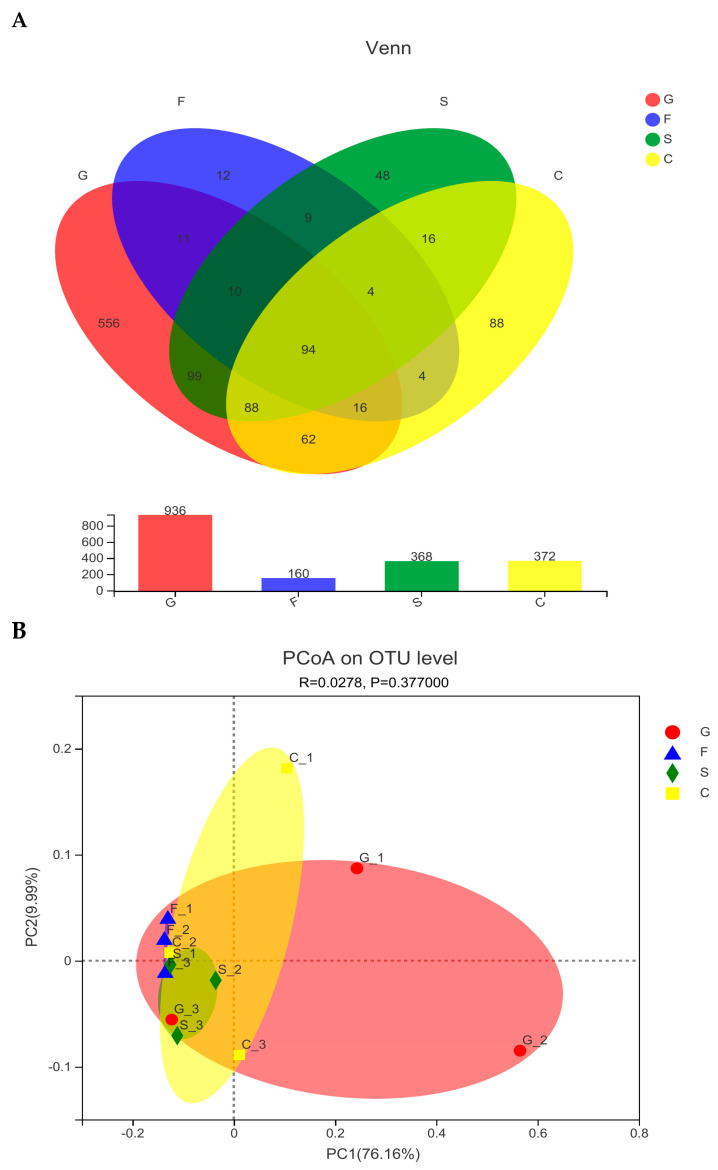
Diversity analysis of intestinal microbiota in *Pelodiscus sinensis* fed with diets containing different types of carbohydrates. (**A**) Venn diagram of intestinal microbial OTUs. (**B**) PCoA analysis based on weighted unifrac distance. Note: G, F, S, and C represent glucose group, fructose group, α-starch group, and cellulose group; the same applies below.

The intestinal microbial diversity is presented in Table 7 and Figure 3. No significant differences were observed in Shannon, Simpson, Chao1, and ACE parameters among all groups (*p* > 0.05). There were 1117 OTUs in all the samples. The total OTUs in the glucose group, fructose group, α-starch group, and cellulose group were 935 OTUs, 156 OTUs, 368 OTUs, and 375 OTUs, respectively (Figure 3A). Principal coordinates analysis (PCoA) showed that the samples in each group were close to each other or were clustered together. No significant difference was found in the diversity of microbial communities among groups (Figure 3B).

**Figure 4:**
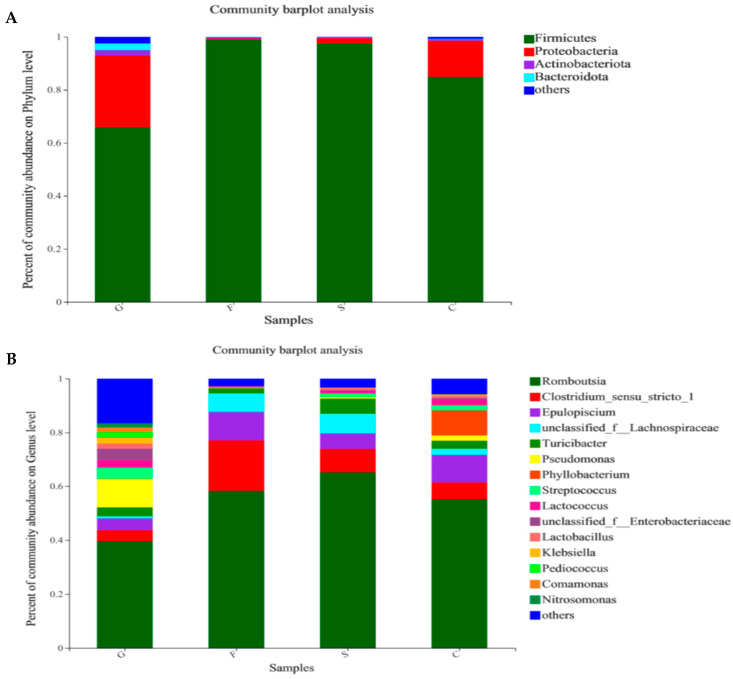
Relative abundances of dominant intestinal microbiota in the intestine of *P. sinensis* fed with diets containing different carbohydrate sources. (**A**) Relative abundance of microbial phyla. (**B**) Relative abundance of microbial genera.

As shown in Figure 4, the dominant intestinal microfloras at the phylum level were Firmicutes, Proteobacteria, and Bacteroidetes. The turtles fed with fructose and α-starch diets showed a higher abundance of Firmicutes and a lower abundance of Proteobacteria compared with those fed with glucose and cellulose diets, and the ratio of Firmicutes to Bacteroides was the largest in the starch group. A higher abundance of Proteobacteria, Actinobacteria, and Bacteroidetes was discovered in the turtles fed with the glucose diet (Figure 4A). At the genus level, *Romboutsia*, *Clostridium-sensu-stricto-1*, and *Epulopiscium* were the dominant microflora. The *Romboutsia* abundance in the starch group was the highest among all groups, while the fructose diet increased the *Clostridium-sensu-stricto-1* and *Epulopiscium* abundance. The *Clostridium-sensu-stricto-1* and *Epulopiscium* abundance in the glucose group was much lower than that in the other groups (Figure 4B).

**Figure 5:**
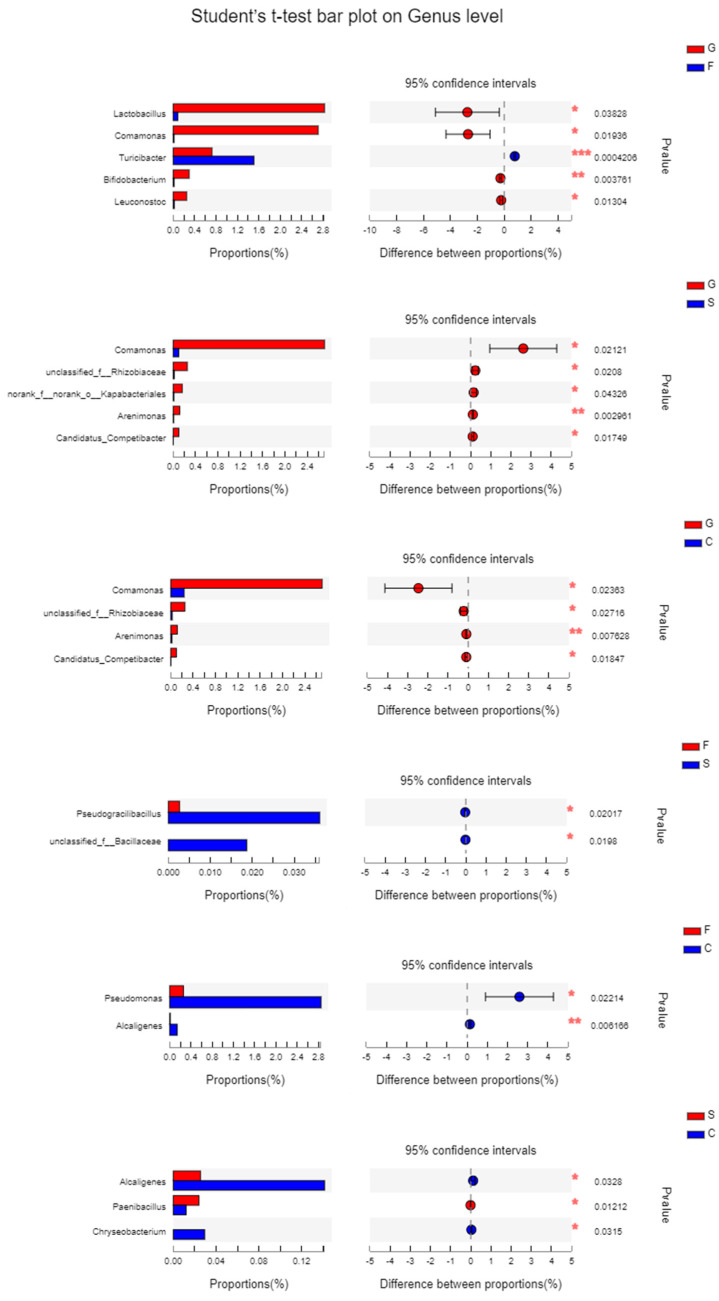
Comparison of microbiota abundances in the intestine of *P. sinensis* fed with diets containing different carbohydrate sources. (**A**) Relative abundance of microbial phyla. (**B**) Relative abundance of microbial genera. * indicates 0.01 < *p* ≤ 0.05, ** indicates 0.001 < *p* ≤ 0.01, *** indicates *p* ≤ 0.001.

Student’s *t*-tests on genus-level taxa were performed to identify the bacterial genera exhibiting significant differences between two groups (Figure 5). The abundances of *Lactobacillus*, *Comamonas*, *Bifidobacterium*, and *Leuconostoc* in the glucose group were significantly higher than those in the fructose group (*p* = 0.038, 0.019, 0.004, and 0.013, respectively), and the *Turicibacter* abundance was significantly lower than that in the fructose group (*p* < 0.001). The *Comamonas* and *Arenimonas* abundances in the starch group (*p* = 0.021 and 0.003, respectively) and the cellulose group (*p* = 0.024 and 0.008, respectively) were significantly decreased compared with the glucose group. The *Psedogracilibacillus* abundances in the starch diet were higher than that in the fructose diet (*p* = 0.020). The *Pseudomonas* and *Alcaligenes* abundances in the cellulose group were significantly higher than those in the fructose group (*p* = 0.022 and 0.006, respectively). The *Paenibacillus* abundance in the cellulose group was significantly lower than that in the starch group (*p* = 0.012). However, the *Alcaligenes* and *Chryseobacterium* abundances were remarkably increased compared with the starch group (*p* = 0.033 and 0.032, respectively).

## 4. Discussion

The present study demonstrated that four types of carbohydrate dramatically impacted the growth performance of *P. sinensis*. The best growth performance and feed efficiency were found in the starch diet, followed by the glucose, fructose, and cellulose groups in sequence. This finding suggests that *P. sinensis* has a better utilization of digestible polysaccharides compared to monosaccharides and indigestible polysaccharides. This is consistent with our previous study on *P. sinensis*, although fructose was not included in it [11]. Conversely, this finding is inconsistent with the results of the red-footed tortoise (*Chelonoidis carbonaria*), a forest-dwelling herbivorous tortoise. No significant difference was observed in the growth of the red-footed tortoise when fed with starch and fiber [31]. Apart from that, there are no other research reports on the utilization of carbohydrate sources in reptiles to date. Because few reptiles have become cultured economic species, the nutritional requirements and metabolic mechanism of reptiles were not well documented [6]. Previous studies have demonstrated that the metabolism characteristics of *P. sinensis* on nutrient utilization are more likely to resemble those of carnivorous fish [4]. Similar results were also observed in gibel carp *Carassius auratus* [16], cobia *Rachycentron canadus* [12], sturgeon *Acipenser schrenckii* [9], Nile tilapia [32], and amur minnow *Rhynchocypris lagowskii* [33]. As a comparison, some fish such as gilthead sea bream *Sparus aurata*, grass carp *Ctenopharyngodon idellus*, and blunt snout bream *Megalobrama amblycephala* [18,19,20], which are mostly herbivorous or omnivorous, preferred monosaccharides to be their optimal carbohydrate source. The different ability to utilize carbohydrate sources among these animals may be directly related to the trophic level and food habits of the species [34], specifically in terms of the diverse gastrointestinal structure, digestive and absorptive system, and metabolism regulations. 

The stunted growth performance of turtles fed with glucose and fructose diets in this study was attributed to the disorder of monosaccharide absorption and metabolism within the body. Upon ingestion, the high concentrations of monosaccharides in the glucose and fructose diets were absorbed quickly and directly into the bloodstream without prior decomposition, resulting in a reduced absorption efficiency and elevated postprandial blood glucose levels. This is supported by the findings of our study, which showed that turtles fed with glucose and fructose diets exhibited significantly lower apparent digestibility coefficients and higher plasma glucose contents compared to those fed with the α-starch diet. Comparable results have also been observed in Nile tilapia [32]. Furthermore, the rapid absorption of monosaccharides is far higher than their utilization rate, which leads to prolonged postprandial hyperglycemia and the abnormal accumulation of liver glycogen [35]. In the current research, the plasma glucose, HSI, and liver glycogen content of turtles fed with monosaccharide diets were significantly higher than those fed with α-starch diet. The higher HSI may be related to the increased glycogen content in the liver [16]. The results of this study suggested that the turtles fed on a monosaccharide diet were more likely to convert excess glucose into glycogen and store it in the liver. Nevertheless, the slow digestion of α-starch was helpful to reduce glucose stress caused by postprandial glucose loading in aquatic animals [36]. The digestible polysaccharides in feed are more easily converted into lipids compared to monosaccharides, as evidenced by the findings that the lipid retention efficiency in the starch group exceeded 150%. In the present study, the lipid contents of the whole body and liver, as well as the plasma triglyceride content, of the turtles fed with an α-starch diet were higher than those fed with glucose and fructose diets. This is consistent with the reports in grouper *Epinephelus malabaricus* [37] and Nile tilapia [32]. Moreover, the accumulation of a large number of red lipid droplets in the liver sections of the turtles in the starch group observed in this study also further confirmed that digestible polysaccharides were preferred to enhance lipid deposition over monosaccharides. At the same time, these results further demonstrated that glucose metabolism exhibited a profound interplay with lipid metabolism, which significantly influencing the utilization ability of carbohydrates with different complexities [38].

The present study showed that the expression of genes involved in glucose metabolism was significantly affected by dietary carbohydrates sources. Compared to turtles fed with a starch diet, those fed with glucose and fructose diets down-regulated the key genes involved in glucose catabolism (such as *pk*), and conversely up-regulated the expression of key gluconeogenesis genes such as *g6pase*, *pepck*, and *fbp*. Similar results were found in the studies of cobia and Nile tilapia [12,17]. These results indicated that turtles fed with a monosaccharide-based diet exhibited limited effectiveness in suppressing gluconeogenesis, ultimately worsening glucose metabolism abnormalities and subsequently leading to a decline in the growth performance and feed utilization rate of the turtles in the monosaccharide groups in this study. 

G6PDH is one of the key enzymes on the pentose phosphate pathway involved in fatty acid synthesis [7,38]. In this study, the expression of *g6pdh* of the turtles fed with a starch diet was higher than those fed with other diets, which is consistent with the results in blunt snout bream, gilthead sea bream, grouper, and Nile tilapia [7,15,17,37]. The glycolytic capacity of the turtles fed with an α-starch diet was also higher than those in the glucose and fructose groups in this study. The dihydroxyacetone phosphate produced in glycolysis can be converted into glycerol 3-phosphate. Acetyl coenzyme A, an important component of lipid synthesis, is generated through the complete dehydrogenation of both glycerol 3-phosphate and pyruvate [39]. Moreover, on the pathway of lipid metabolism, the expression of the genes involved in lipid metabolism (except for *acaca*) were up-regulated by stimulating the expression of the *srebp* and *chrebp* genes in the livers of turtles fed with a starch diet, which indicated that the glucolipid conversion efficiency of the turtles fed with an α-starch diet was higher than that of the turtles fed with other diets. The increased expression of lipolysis-related genes (*pparα*, *acox1*, and *cpt*) in starch diets could reduce the risk of diseases caused by excessive fat deposition, thereby ensuring the health of the turtles. This also provides a valid molecular explanation for the above physiological observation that digestible polysaccharides are more easily converted into lipids than monosaccharides.

Fructose is an isomer of glucose that is found in nature and can be converted into glucose within the body. However, fructose metabolism differs from glucose metabolism in that it is not regulated by insulin. In mammals, fructose is often considered as the primary trigger for inducing non-alcoholic fatty liver disease due to its tendency to promote lipid synthesis [40,41]. Fructose has been demonstrated to facilitate triglycerides and lipid synthesis, as well as elevate the mRNA expression of the enzymes involved in glycolysis, lipogenesis, and gluconeogenesis [42,43]. Nevertheless, contrary to expectations, our study did not reveal this phenomenon in *P. sinensis*. Instead, we observed that the lipid droplets, the LRE values, and the crude lipid contents of whole body and liver were significantly lower in turtles fed with fructose compared to those fed with glucose. Additionally, the expressions of key genes on the pathway of lipid metabolism in the liver were down-regulated in the fructose group compared to the glucose group, including the expression of *srebp* and *chrebp*. Based on these observations, it is speculated that fructose may interfere with normal lipid metabolism by inhibiting the expression of the *srebp* and *chrebp* genes of the turtles. This finding aligns with reports on other aquatic animals, such as sturgeon and Nile tilapia [16,17]. Up until now, no study has reported that fish can efficiently utilize fructose like glucose, even in the case of pacu (*Piaractus mesopotamicus*), a species of fruit-eating fish [44]. Therefore, the metabolic characteristics of *P. sinensis* on fructose utilization are more akin to those of fish, and they cannot utilize fructose as a suitable carbohydrate source. Actually, fructose primarily comes from fruits in nature, but fruits are not part of the dietary preferences of *P. sinensis*. It is conceivable that the turtle’s inefficient utilization of fructose in this study stems from its long-term adaptive evolution in an aquatic habitat where fructose sources are relatively scarce.

Cellulose is an indigestible carbohydrate for most animals except for herbivores. In this experiment, cellulose was included as a negative control to evaluate the growth and physiological responses of turtles when fed zero-digestible carbohydrate diets. As anticipated, the results of this study clearly showed that *P. sinensis* lacked the ability to digest cellulose. The lowest ADC in the cellulose group is closely related to the shorter villi observed in the histomorphological sections. All key genes involved in glucose and lipid metabolism were inactive compared to other carbohydrate sources, which resulted in the lowest digestibility, growth, feed utilization, and lipid deposition among all groups. Consequently, cellulose did not serve as an energy source for *P. sinensis*.

The dietary component is an important factor influencing the diversity and structure of the intestinal microbiota [45]. In this study, carbohydrate sources did not affect the diversity and richness of intestinal microbiota. At the phylum level, Firmicutes, Proteobacteria, and Bacteroidetes were the dominant intestinal bacterial species in *P. sinensis*, and similar patterns had been observed in other animal species. For example, the cecal microbiota of mice fed with dietary carbohydrates was predominantly composed of Firmicutes, and contained low levels of Bacteroidetes and Actinobacteria [46]. Firmicutes and Bacteroides play an important role in host lipid metabolism, and their ratios are proportional to the ability of the host to store lipid [47]. In this study, the ratio of Firmicutes to Bacteroides was the largest in the intestinal microbiota of the α-starch group. This might be the reason why the α-starch group had advantages in growth and lipid metabolism. At the genus level, the probiotic content (*Paenibacillus*) of turtles in the cellulose group was lower, while the content of opportunistic pathogens (*Pseudomanas* and *Chryseobacterium*) was increased compared to other groups. *Pseudomonas* and *Chryseobacterium* were common pathogenic bacteria in most aquaculture animals [48]. The high abundance of *Chryseobacterium* might affect the growth and survival of aquatic animals [49,50,51]. The overgrowth of opportunistic pathogens may be due to the damage of the intestinal microbiota barrier caused by the defect of the host immune defense system or intestinal mucosal barrier [52]. The results in this study suggest that the long-term consumption of excessive cellulose (17%) will threaten the intestinal health of the turtles. To the best of our knowledge, this is the first report on the relationship between carbohydrate sources and intestinal microbiota in soft-shelled turtles. Intestinal microbiota may be an important indicator for monitoring nutritional status and physical health, and should not be ignored.

## 5. Conclusions

**Figure 6:**
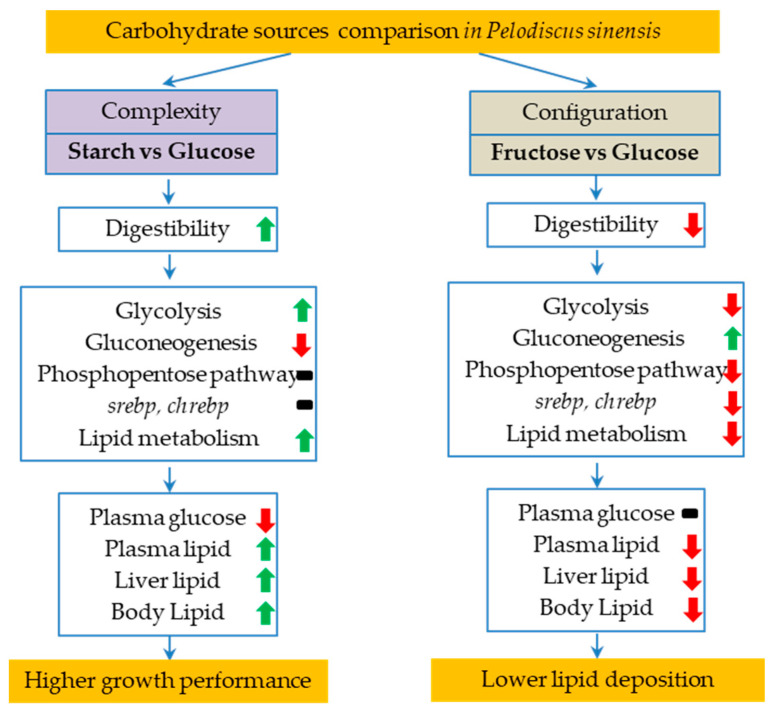
General summary for the comparative responses in *P. sinensis* between starch and glucose diets, as well as fructose and glucose diets. The green arrow indicates the promoting effect; the red arrow indicates the decreasing effect; the black horizontal line indicates no effect.

Overall (Figure 6), the soft-shelled turtle exhibited a superior utilization of starch compared to glucose and fructose. Dietary starch has a strong lipogenic function by inducing the expression of the genes involved in glucolipid metabolism, with the results of elevated plasma TG levels and increased lipid contents in both the whole body and the liver. Glucose and fructose diets caused postprandial hyperglycemia in *P. sinensis* due to the un-inhibited gluconeogenesis. *P. sinensis* fed with fructose did not show a higher capacity for lipid deposition than glucose, as seen from mammals. Instead, fructose interfered with the glucolipid metabolism of *P. sinensis* by suppressing the expression of *shrebp* and *chrebp*. Cellulose did not serve as an energy source for *P. sinensis*.

## Figures and Tables

**Table 1:** Formulation and proximate composition of experimental diets (air-dried basis).

Ingredients (g/kg)	Glucose	Fructose	Starch	Cellulose
White fishmeal ^a^	480	480	480	480
Gelatin ^b^	40	40	40	40
Casein ^b^	110	110	110	110
Squid liver meal ^a^	50	50	50	50
Glucose ^b^	170	0	0	0
Fructose ^b^	0	170	0	0
α-cassava-starch ^a^	0	0	170	0
Microcrystalline cellulose ^b^	0	0	0	170
Phospholipid ^a^	10	10	10	10
Soybean oil ^a^	40	40	40	40
CaHPO_4_^a^	15	15	15	15
Limestone powder ^a^	8	8	8	8
Zeolite powder ^a^	54	54	54	54
Choline chloride ^a^	2	2	2	2
Yttrium oxide ^b^	1	1	1	1
Premix ^c^	20	20	20	20
Proximate composition (% dry matter)				
Crude protein	48.80	48.58	47.42	47.09
Crude lipid	7.84	7.89	7.84	7.80
Crude ash	21.00	21.38	20.33	20.68
Gross energy (MJ/kg)	24.30	25.17	24.84	24.67

^a^ Purchased from Hebei Haitai Technology Co., Ltd. (Shijiazhuang, China). ^b^ Purchased from Shijiazhuang Ningnuo Co., Ltd. (Shijiazhuang, China). ^c^ Premix: VB_1_, 0.5 mg/g; VB_2_, 1.2 mg/g; VB_3_, 3.0 mg/g; VB_5_, 5.0 mg/g; VB_6_, 1.2 mg/g; VB_11_, 0.1 mg/g; VB_12_, 1.0 μg/g; biotin, 2.0 μg/g; inositol, 15.0 mg/g; VC, 20.0 mg/g; VA, 1000 IU/g; VD_3_, 500 IU/g; VE, 5.5 mg/g; VK_3_, 0.5 mg/g; CuSO_4_•5H_2_O, 0.5 mg/g; ZnSO_4_•H_2_O, 5.0 mg/g; FeSO_4_•7H_2_O, 8.0 mg/g; MnSO_4_•H_2_O, 3.0 mg/g; Na_2_SeO_3_•5H_2_O, 20 μg/g; CoCl•6H_2_O, 50 μg/g; KI, 40 μg/g. All ingredients used in the premix were purchased from Hebei Haitai Technology Co., Ltd. (Shijiazhuang, China).

**Table 2:** Nucleotide sequences of primers used to quantify gene expressions in the real-time PCR analysis.

Gene	Forward Primer (5′-3′)	Reverse Primer (5′-3′)	Accession Number
*glut2*	GGACTTGTTCTCCTGACCACT	CCAGTTGCAGAACCCAGCTA	XM_006122851
*hk*	ACTCTGTCACTAGCCCCTGT	AGCCAGAGCACCGATACAAC	XM_006121526
*pk*	GAACCGGGATCCTACTGAAGC	TCGTGCCTTGCCAACATTC	XM_006127865
*pfk*	CCGGCTACGTGAAGGATCTG	TTCTCGTCCATCGCCTTCTG	KF944472
*g6pase*	GTCTGCTTGTCCCGAGTCTTC	TCCGCTACTACCATCCCTGAGA	XM_006125698
*pepck*	ATAGGCAGGGGGCTATGCTG	TGCGCATGTAACTGTGTGAGA	XM_006125340
*fbp*	GTTTCCTCTGGGGAACCGTC	GAATTCCCCAATTGCCGGGT	XM_006127656
*gys*	CCTAGGGGAGTTCACTCAGGA	AGCATCGGGTTGTTCAGTCA	XM_006125340
*pyg*	CGGCATCTGGACAAAGTTGC	TGGATCTTTGCTACGCCGTT	XM_006116303
*g6pdh*	GAGGTGCGGCTACAGTTCC	CGATTGCCATAGGTCAGGT	XM_006130063
*fasn*	GTCTGTCCACGGCCGATATT	ACGACGTCACAAGCTTCAGT	XM_025178574
*acaca*	CCTCTAACCCAAAGCCCCTG	CCGTAACGCTAGACAGTCCC	XM_014581353
*ppar* *α*	CTTGCACTTTGGTCCCTCCT	AGGCCACAAAGGATCACTGG	XM_025187026
*acox1*	ATGAGCCCTTGCCAGGTATT	CGGGGAATGCGAAAGTTGTC	XM_006136519
*cpt1*	GCCGTTATTTCAAAGTCTGTC	ATCCTCTTCCCTGTATCCCT	XM_006131643
*srebp*	TATTGGTCCATTTCTCCAGCCTTCT	CCAGCACTAACACTGAGCCCTTC	XM_025186382
*chrebp*	GTGTCTTCCCAGCTCACCAA	GAATGGGGTCCAGAGTGTCC	XM_006113748
*β-actin*	GCTACGTTGCCCTGGATTTTG	CATACCCAGGAAGGATGGCT	XM_006134860

*glut2*, glucose transporter 2; *hk*, hexokinase; *pk*, pyruvate kinase; *pfk*, fructose phosphokinase; *g6pase*, glucose-6-phosphatase; *pepck*, phosphoenolpyruvate carboxykinase; *fbp*, fructose-1,6-bisphosphatase; *gys*, glycogen synthase; *pyg*, glycogen phosphorylase; *g6pdh*, glucose-6-phosphate dehydrogenase; *fasn*, fatty acid synthase; *acaca*, acetyl-CoA carboxylase; *pparα*, peroxisome proliferator-activated receptor; *acox1*, acyl-CoA oxidase 1; *cpt1*, carnitine palmitoyl transferase I; *srebp*, sterol regulatory element-binding transcription factor; *chrebp*, carbohydrate response element binding protein.

**Table 3:** Growth performance of *Pelodiscus sinensis* fed with diets containing different types of carbohydrate (*n* = 7).

	Glucose	Fructose	Starch	Cellulose
IBW (g)	5.17 ± 0.01	5.14 ± 0.01	5.15 ± 0.01	5.15 ± 0.02
FBW (g)	26.03 ± 1.54 ^ab^	25.23 ± 1.15 ^ab^	29.10 ± 1.68 ^b^	23.48 ± 0.81 ^a^
FR (%/d)	2.10 ± 0.04 ^b^	2.18 ± 0.03 ^b^	1.83 ± 0.02 ^a^	2.10 ± 0.03 ^b^
WGR (%)	403.65 ± 30.55 ^ab^	398.51 ± 25.26 ^ab^	465.40 ± 32.05 ^b^	355.74 ± 14.76 ^a^
SGR (%/d)	2.68 ± 0.10 ^ab^	2.67 ± 0.09 ^ab^	2.87 ± 0.09 ^b^	2.58 ± 0.05 ^a^
FCR	0.95 ± 0.02 ^b^	0.99 ± 0.02 ^b^	0.79 ± 0.01 ^a^	0.99 ± 0.01 ^b^
PER (%)	211.19 ± 3.62 ^a^	207.82 ± 3.29 ^a^	252.41 ± 1.73 ^b^	206.19 ± 3.65 ^a^
PDE (%)	45.45 ± 0.85 ^c^	41.97 ± 0.52 ^b^	67.34 ± 0.49 ^d^	36.74 ± 0.83 ^a^
LDE (%)	100.26 ± 1.62 ^c^	69.34 ± 0.82 ^b^	151.96 ± 0.90 ^d^	49.11 ± 1.25 ^a^
HSI (%)	4.74 ± 0.17 ^b^	4.67 ± 0.11 ^b^	4.16 ± 0.13 ^a^	3.97 ± 0.10 ^a^
VSI (%)	9.39 ± 0.16 ^b^	9.10 ± 0.22 ^b^	8.25 ± 0.21 ^a^	8.94 ± 0.13 ^b^

Values in the same row followed by different superscripts letters are significantly different (*p* < 0.05). IBW, initial body weight; FBW, final body weight; FR, feeding rate (%/d) = 100 × total dry feed intake/(days × (IBW + FBW)/2); WGR, weight gain rate (%) = 100 × (FBW − IBW)/IBW; SGR, specific growth rate (%/d) = 100 × (Ln (FBW) − Ln (IBW))/days; FCR, feed conversion ratio = dry feed intake/(FBW − IBW); PER, protein efficiency ratio (%) = 100 × ((FBW − IBW)/protein intake); PDE, protein deposition efficiency (%) = 100 × protein retained in body/protein intake; LDE, lipid deposition efficiency (%) = 100 × lipid retained in body/lipid intake; HSI, hepatic somatic index (%) = 100 × (liver weight/body weight); VSI, visceral somatic index (%) = 100 × (visceral weight/body weight).

**Table 4:** Apparent digestibility coefficients of *Pelodiscus sinensis* fed with diets containing different types of carbohydrate (%) (*n* = 3).

	Glucose	Fructose	Starch	Cellulose
ADC_dry matter_	79.26 ± 0.11 ^b^	78.06 ± 0.22 ^c^	80.99 ± 0.30 ^a^	66.91 ± 0.19 ^d^
ADC_protein_	93.85 ± 0.03 ^a^	93.38 ± 0.07 ^b^	93.81 ± 0.10 ^a^	93.34 ± 0.04 ^b^
ADC_carbohydrate_	80.65 ± 0.10 ^b^	72.56 ± 0.28 ^c^	82.20 ± 0.28 ^a^	8.17 ± 0.52 ^d^
ADC_energy_	92.95 ± 0.10 ^b^	92.16 ± 0.12 ^c^	93.41 ± 0.10 ^a^	84.06 ± 0.14 ^d^

Values in the same row followed by different superscripts letters are significantly different (*p* < 0.05). ADC represents apparent digestibility coefficient.

**Table 5:** Proximate compositions of whole body and liver of *Pelodiscus sinensis* fed with diets containing different types of carbohydrate (wet-weight basis, %) (*n* = 7).

	Glucose	Fructose	Starch	Cellulose
Whole body				
Moisture	65.56 ± 1.68 ^b^	69.36 ± 0.99 ^c^	58.72 ± 1.08 ^a^	72.95 ± 0.82 ^d^
Crude protein	20.28 ± 0.53 ^c^	18.92 ± 0.33 ^b^	24.33 ± 0.41 ^d^	17.00 ± 0.25 ^a^
Crude lipid	6.73 ± 0.32 ^c^	5.01 ± 0.24 ^b^	8.32 ± 0.19 ^d^	3.68 ± 0.13 ^a^
Crude Ash	6.19 ± 0.15 ^b^	5.82 ± 0.07 ^bc^	6.82 ± 0.16 ^a^	5.39 ± 0.18 ^c^
Liver				
Moisture	63.10 ± 0.41	63.49 ± 1.16	62.46 ± 2.44	61.62 ± 0.42
Lipid	15.14 ± 0.51 ^b^	10.92 ± 1.02 ^a^	15.76 ± 1.01 ^b^	11.90 ± 0.70 ^a^
Glycogen (mg/g)	25.04 ± 1.08 ^b^	23.09 ± 1.38 ^b^	22.96 ± 1.05 ^b^	15.21 ± 1.60 ^a^

Values in the same row followed by different superscripts letters are significantly different (*p* < 0.05).

**Table 6:** Plasma biochemical parameters of *Pelodiscus sinensis* fed with diets containing different types of carbohydrate (*n* = 7).

	Glucose	Fructose	Starch	Cellulose
GLU (mmol/L)	7.44 ± 0.57 ^b^	7.39 ± 0.83 ^b^	5.23 ± 0.64 ^a^	5.78 ± 0.89 ^a^
TG (mmol/L)	3.93 ± 0.20 ^b^	1.39 ± 0.19 ^a^	4.52 ± 0.30 ^c^	0.94 ± 0.15 ^a^
CHOL (mmol/L)	7.91 ± 0.20 ^a^	8.84 ± 0.34 ^b^	8.97 ± 0.34 ^b^	7.63 ± 0.17 ^a^
TP (g/L)	30.10 ± 1.11	32.69 ± 1.24	30.68 ± 1.21	29.99 ± 1.19

Values in the same row followed by different superscripts letters are significantly different (*p* < 0.05). GLU, glucose; TG, triacylglycerol; CHOL, total cholesterol; TP, total protein.

**Table 7:** Diversity indexes on operational taxonomic units (OTUs) level in intestinal microbiota of *Pelodiscus sinensis* fed with diets containing different carbohydrate sources.

	Glucose	Fructose	Starch	Cellulose
Shannon	3.58 ± 0.21	1.30 ± 0.36	1.41 ± 0.56	1.80 ± 0.28
Simpson	0.105 ± 0.04	0.43 ± 0.15	0.46 ± 0.13	0.36 ± 0.08
Chao	596.99 ± 252.1	102.1 ± 68.28	260.02 ± 123.49	214.65 ± 103.83
ACE	596.65 ± 60.88	104.45 ± 74.05	357.14 ± 198.25	262.42 ± 58.07
Coverage	99.89 ± 0.02	99.93 ± 0.05	99.84 ± 0.10	99.90 ± 0.06

## Data Availability

Data are contained within the article.
